# Impact of tourniquet on short-term outcomes in opening wedge high tibial osteotomy with modern tranexamic acid protocols: a retrospective cohort study

**DOI:** 10.1186/s12891-021-04830-4

**Published:** 2021-11-08

**Authors:** Limin Wang, Zhen Zhang, Wei Xiong, Qian Fang, Yunfeng Tang, Guanglin Wang

**Affiliations:** Department of Orthopaedics, West China Hospital, West China School of Medicine, Sichuan University, No. 37, Wuhou Guoxue Road, 610041 Chengdu, Sichuan P. R. China

**Keywords:** High tibial osteotomy, Tourniquet, Tranexamic acid

## Abstract

**Background:**

The use of a tourniquet during high tibial osteotomy (HTO) is a routine procedure, but there is currently no research on the benefits and potential risks of tourniquet use during HTO. The aim of this study was to investigate the impact of tourniquet on perioperative blood loss, early functional recovery and complications in opening wedge HTO with modern tranexamic acid protocols.

**Methods:**

This was a retrospective cohort study of patients who underwent unilateral opening wedge HTO between January 2019 and September 2020. All patients were divided into two groups according to whether a tourniquet was applied during HTO. Patients in both groups received the same surgical procedures, tranexamic acid protocols and other perioperative treatments. Preoperative baseline data, intraoperative data, early postoperative recovery and all complications during the 3-month follow-up were collected and compared between the two groups.

**Results:**

A total of 62 patients were enrolled in this study, including 32 in the tourniquet group and 30 in the non-tourniquet group. There was no significant difference in preoperative baseline data between the two groups (*P* > 0.05 in all). Intraoperative blood loss in the tourniquet group was significantly lower than that in the non-tourniquet group (80.22 ml versus 94.00 ml, *P* < 0.001), but there was no difference in total blood loss (187.39 ml versus 193.31 ml, *P* = 0.714). And no patient in either group required blood transfusion. In terms of early postoperative recovery, tourniquet use significantly increased pain scores and reduced knee range of motion on the first and second postoperative days, but there was no significant difference between the two groups at postoperative third day and third month. Furthermore, there was no significant difference between the two groups in terms of lower limb force line correction, length of stay, Knee Society Score or the incidence of complications during the 3-month follow-up (*P* > 0.05 in all).

**Conclusions:**

In opening wedge HTO with modern tranexamic acid protocols, not using a tourniquet does not increase perioperative total blood loss or the risk of complications, but facilitates early postoperative recovery by reducing pain and increasing range of motion.

## Background

High tibial osteotomy (HTO) is a well-established surgical procedure for active patients with medial unicompartmental osteoarthritis (OA) and varus alignment deformity of the knee joint [1]. It has been reported that HTO can effectively prevent or inhibit the progression of OA of the knee and avoid or postpone knee replacement for patients with medial knee OA [2]. During the HTO procedure, surgeons are accustomed to using a tourniquet to reduce intraoperative bleeding at the osteotomy site, thereby exposing the surgical field more clearly and possibly reducing blood transfusion rates [3]. However, tourniquet inflation and deflation can cause ischaemia-reperfusion injury, which increases local oxidative stress and the inflammatory response [4]. Many studies have shown that tourniquet use is associated with nerve palsies [5], thigh pain [6, 7], muscle necrosis [8], rhabdomyolysis [9], decreased quadriceps muscle strength [10] and increased risk of venous thromboembolism [11]. In addition, recent studies have shown that tourniquet use, while reducing intraoperative bleeding, does not significantly reduce postoperative total blood loss or blood transfusion rates [12, 13]. This phenomenon may be a result of increased hidden blood loss due to hyperfibrinolysis following tourniquet release [14].

Currently, tranexamic acid (TXA) has become a routine strategy for perioperative blood management in orthopaedic surgery. As an antifibrinolytic solvent, TXA can block the lysine binding site on plasminogen, thereby inhibiting the formation of plasmin, thus stabilizing blood clots and reducing bleeding [15]. Currently, many studies [16–19] have confirmed that TXA can significantly reduce intraoperative and postoperative bleeding during HTO without increasing the risk of complications. In addition, several studies have reported that forgoing routine use of tourniquets in total knee arthroplasty (TKA) using modern TXA protocols does not increase total blood loss, but facilitates rapid recovery [20, 21]. However, there is a paucity of data that explores the effect of tourniquet use in HTO with modern TXA protocols.

Therefore, the aim of this study was to evaluate the influence of tourniquet use in HTO with modern TXA protocols on perioperative blood loss, early functional recovery, and the risk of complications.

## Materials and methods

### Patient selection

This was a retrospective cohort study of 81 patients who underwent unilateral opening wedge HTO between January 2019 and September 2020. The inclusion criteria of this study were (1) diagnosis of primary medial unicompartmental knee OA with varus deformity, (2) stable knee without ligament insufficiency, (3) no pain relief after 3 months of conservative treatment, and (4) age between 21 and 69 years. Patients who met the following criteria were excluded: history of deep venous thrombosis (DVT) or pulmonary embolism (PE), myocardial infarction or stroke within 6 months prior to surgery, renal dysfunction, peripheral vascular disease, body mass index (BMI) of > 35 kg/m^2^, bilateral HTO, simultaneous femoral osteotomy, or follow-up of less than 3 months. Due to concerns regarding the safety of tourniquet use, the senior author discontinued the routine use of tourniquets in HTO since November 2019, which provided us with an opportunity to study the effect of tourniquets on postoperative outcomes. All patients were divided into two groups according to whether a tourniquet was applied during HTO, namely the tourniquet group (January 2019–November 2019) and the non-tourniquet group (November 2019–September 2020). Patients in both groups received the same perioperative care protocols. This retrospective study was approved by the institutional review board of the authors’ institution. Written informed consents before participation were given by participants before the initial assessment.

### Surgical procedures

Preoperative planning was carried out using the Dugdale method as described in the literature [22]. All opening wedge HTOs were performed, with the patients under general anaesthesia, by the same surgeon. A tourniquet was placed at the root of the thigh for all patients. For the tourniquet group, the tourniquet was inflated to 100 mmHg above systolic pressure from incision until the leg was wrapped with elastic bandages. For the non-tourniquet group, the tourniquet was not inflated during surgery. Through an anteromedial longitudinal skin incision of approximately 5 cm, the superior border of the pes anserinus and the superficial medial collateral ligament were partially released, and the posterior neurovascular elements were retracted by a Hohmann retractor. A biplanar osteotomy was performed with the positioning of Kirschner wires [23]. The gap was opened gradually until the target mechanical axis was obtained. During the procedure, caution should be exercised to avoid fracture of the lateral tibial cortex. The osteotomy site was fixed with a TomoFix locking plate and locking screws. No bone graft or bone substitute was placed in the osteotomy site. No drainage tube was placed at the wound site in all patients.

### Perioperative management

All patients received the same perioperative TXA protocol, including 2 g of TXA intravenously before tourniquet inflation and another 2 g administered at 3 h postoperatively. Cefuroxime 1.5 g was used for antibiotic prophylaxis 30 min before skin incision. All patients received a patient-controlled analgesia pump within 24 h after surgery and 200 mg of celecoxib was given orally every 12 h from postoperative Day 1 to Day 14. To prevent thrombosis, enoxaparin (Clexane; 0.4 mL containing 4000 IU) was injected subcutaneously once a day from 12 h after the operation until discharge. In addition, all patients were encouraged to perform isometric quadriceps femoris contraction training on the postoperative day. From the first postoperative day, passive full-range knee flexion and extension exercises were performed, and partial weight-bearing walking was performed with the assistance of crutches. All patients began full weight-bearing walking at 3 weeks postoperatively.

### Outcome measurement

In order to minimize bias, data collectors and data analysts were blinded to the study design in this study. Preoperative data including sex, age, height, weight, BMI, American Society of Anaesthesiologists (ASA) class, surgical site, preoperative haemoglobin (Hb) and haematocrit (Hct), range of motion (ROM), visual analogue scale (VAS) for pain, and Knee Society Score (KSS) were collected from electronic medical records and our follow-up database. At the same time, the following indicators, including the OA grading (Kellgren-Lawrence grade [24]), medial proximal tibia angle (MPTA), hip-knee-ankle angle (HKA), and posterior tibial slope were measured in the preoperative standing anteroposterior view, true lateral view, and weight-bearing whole-leg radiographs, according to the methods described in the literature [25]. The KSS [26] includes two subscores: the knee score and the function score. In each subscore, 100 points represent the best knee function.

The intraoperative characteristics to be collected included the use of a tourniquet, operation time (beginning at incision until the completion of the wound closure), intraoperative blood loss, and number of radiological exposures. The intraoperative blood loss was equal to the increased weight of the gauze plus the volume of the aspirator bottle excluding rinsing fluid. Postoperatively, decreases in Hb and Hct values (preoperative Hb and Hct values minus postoperative Day 2 Hb and Hct values), total blood loss volume, transfusion rate, MPTA, HKA, posterior tibial slope, and length of stay (LOS) were recorded. In addition, VAS pain scores and ROM of the knee joint were recorded 1, 2, and 3 days after surgery. The VAS pain scores recorded by nursing staff on a 10-point scale (ranging from none to severe) every 4 h were averaged to derive an overall pain score at 1, 2, and 3 postoperative days. Total blood loss was calculated using the validated Gross formula, which included sex, height, and weight, as well as decreases in Hct values with correction for any volume of blood administered [27]. The indications for blood transfusion in our institution were Hb less than 7 g/dL or Hb between 7 and 10 g/dL with obvious signs and symptoms of anaemia (light-headedness, presyncope, fatigue precluding participation in physical therapy, palpitation, or shortness of breath not due to other causes). During outpatient follow-up at 3 months postoperatively, we collected the VAS pain scores and ROM and KSS scores.

In terms of complications, we collected all related complications within 3 months after surgery, including surgical complications and medical complications. Surgical complications included incision complications (haematoma, delayed healing, and superficial or deep wound infection), fracture of the lateral tibial cortex, postoperative nerve palsies and the need for revision. Medical complications include symptomatic DVT, symptomatic PE, myocardial infarction, cerebrovascular accident, gastrointestinal bleeding and pulmonary infections.

### Statistical analyses

Continuous variables are presented as the means with standard deviations, whereas categorical data are shown as frequencies or percentages. Differences in continuous variables between groups were evaluated with Independent-Sample T test or Mann-Whitney U test, depending on the distribution characteristics of the data. A chi-square test or Fisher’s exact test was used to estimate the differences between groups in categorical variables. Values of *P* < 0.05 were considered to be statistically significant. Statistical analysis was performed using SPSS Statistics version 26.0 (IBM).

## Results

Of the 81 patients who underwent opening wedge HTO, 19 met the exclusion criteria and were not included in this study. A total of 62 patients were eventually included in our study, including 32 patients in the tourniquet group and 30 patients in the non-tourniquet group. There were no statistically significant differences between the two groups in sex, age, BMI, ASA class, surgical site, preoperative Hb and Hct, ROM, VAS pain score, KSS score, Kellgren-Lawrence grade, MPTA, HKA or posterior tibial slope. The preoperative characteristics of these patients are presented in Table 1.

**Table 1:** Preoperative baseline characteristics

	Tourniquet group	Non-tourniquet group	***P*** value
Total number of patients	32	30	
Male/female	14/18	14/16	0.818
Age, years	47.91 ± 11.74	46.93 ± 12.30	0.783
Height, cm	160.25 ± 9.00	160.93 ± 7.27	0.744
Weight, kg	67.25 ± 12.60	67.03 ± 16.58	0.842
BMI, kg/m^2^	26.25 ± 4.95	25.70 ± 5.71	0.777
ASA class, n			1.000
1	12	10	
2	12	14	
3	8	6	
Site, right/left	14/18	13/17	0.974
ROM, °	126.88 ± 9.31	127.33 ± 9.80	0.848
VAS	4.03 ± 1.28	4.00 ± 1.31	0.913
KSS			
Knee score	56.84 ± 4.28	55.80 ± 5.14	0.380
Function score	60.13 ± 7.84	59.00 ± 8.35	0.586
Kellgren-Lawrence grade			0.842
1	6	4	
2	14	15	
3	12	11	
MPTA, °	83.82 ± 3.08	84.65 ± 1.47	0.381
HKA angle, °	173.25 ± 1.98	172.82 ± 1.98	0.463
Posterior tibial slope, °	9.38 ± 2.65	10.09 ± 2.03	0.309
Hb, g/dL	140.72 ± 8.56	144.07 ± 10.26	0.070
Hct, %	0.42 ± 0.04	0.43 ± 0.03	0.208

Abbreviations: *BMI* body mass index, *ASA* American Society of Anaesthesiologists, *ROM* range of motion, *VAS* visual analogue scale, *KSS* Knee Society Score, *MPTA* medial proximal tibia angle, *HKA* hip-knee-ankle angle, *Hb* haemoglobin, *Hct* haematocrit, *°* degrees

We found that the average intraoperative blood loss was 80.22 ml in the tourniquet group and 94.00 ml in the non-tourniquet group, and the difference between these two groups was significant (*P* < 0.001). However, there was no significant difference in total blood loss or drop in Hb and Hct values between these two groups. No patient in either group required blood transfusion. In addition, the tourniquet group and non-tourniquet group were similar in terms of operation time, number of radiological exposures, MPTA, HKA, posterior tibial slope and LOS (Table 2).

**Table 2:** Perioperative and postoperative follow-up data

	Tourniquet group	Non-tourniquet group	***P*** value
Surgical duration, min	86.09 ± 14.07	88.23 ± 8.72	0.188
Intraoperative blood loss, ml	80.22 ± 16.82	94.00 ± 14.27	< 0.001
Total blood loss, ml	187.39 ± 73.94	193.31 ± 72.56	0.714
Transfusions, n	0	0	
Decreases in Hb values, g/dL	13.50 ± 5.49	13.17 ± 2.00	0.949
Decreases in Hct values, %	0.05 ± 0.01	0.05 ± 0.02	0.106
Number of radiological exposures	27.16 ± 9.11	26.50 ± 8.00	0.977
MPTA, °	91.29 ± 1.82	91.95 ± 3.31	0.572
HKA angle, °	182.14 ± 2.64	181.10 ± 2.53	0.178
Posterior tibial slope, °	9.92 ± 3.39	10.71 ± 4.16	0.397
KSS at 3 months after surgery			
Knee score	78.59 ± 7.10	80.50 ± 7.11	0.269
Function score	83.34 ± 3.92	84.10 ± 3.21	0.367
LOS, days	7.41 ± 3.37	6.60 ± 1.33	0.396
**Surgical complications**			
Haematoma	0	0	–
Delayed wound healing	1	0	1.000
Superficial or deep wound infection	0	0	–
Fracture of the lateral tibial cortex	6	4	0.815
Need for revision	0	0	–
**Medical complications**			
Symptomatic DVT	0	0	–
Symptomatic PE	0	0	–
Myocardial infarction	0	0	–
Cerebrovascular accident	0	0	–
Gastrointestinal bleeding	0	0	–
Pulmonary infections	0	0	–
**Total complications**	7	4	0.379

Abbreviations: *Hb* haemoglobin, *Hct* haematocrit, *MPTA* medial proximal tibia angle, *HKA* hip-knee-ankle angle, *KSS* Knee Society Score, *LOS* length of stay, *DVT* deep venous thromboembolism, *PE* pulmonary embolisms, *°* degrees

**Fig. 1:**
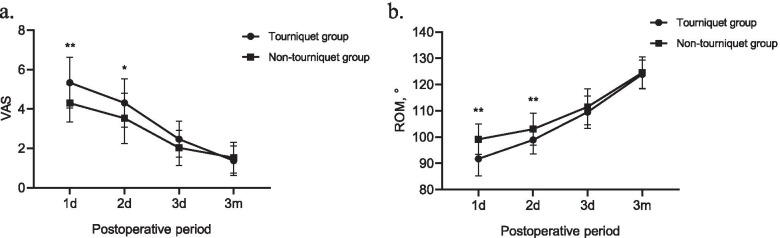
The VAS score and ROM of the knee during early postoperative follow-up. VAS, visual analogue scale; ROM, range of motion. *, *P* < 0.05; **, *P* < 0.01

In terms of early postoperative recovery, tourniquet use significantly increased VAS pain scores on the first and second postoperative days, but there was no significant difference between the two groups at postoperative third day and third month (Fig. 1a). In addition, tourniquet use resulted in significantly reduced knee ROM on the first and second postoperative days, but did not significantly affect knee ROM on the third day and third month after the operation (Fig. 1b). Moreover, the KSS scores at 3 months after surgery in both groups were significantly higher than preoperative KSS scores, although there was no significant difference between the two groups (Table 2).

Furthermore, there was no significant difference in the incidence of overall complications between the two groups during the 3-month follow-up period (21.88% versus 13.33%, *P* = 0.379). One patient in the tourniquet group had delayed wound healing during the follow-up period. There was no significant difference in the incidence of fracture of the lateral tibial cortex between the tourniquet and non-tourniquet groups (18.75% versus 13.33%, *P* = 0.815). No other surgical complications or medical complications occurred during the follow-up period.

## Discussion

To our knowledge, this study is the first to assess the impact of tourniquet application in HTO with modern tranexamic acid protocols on perioperative blood loss, early postoperative recovery, and complications. We found that abandoning routine use of tourniquets during HTO significantly increased intraoperative blood loss, but had no effect on total blood loss and blood transfusion rate and did not increase operative time or number of radiological exposures. In the early postoperative period, patients in the non-tourniquet group had significantly better knee pain relief and ROM than those in the tourniquet group, but there was no significant difference in knee pain, functional recovery or the incidence of complications at 3 months after surgery between these two groups.

For patients who have medial compartmental OA with varus deformity of the knee, high tibial osteotomy, which corrects the limb deformity by shifting the mechanical axis to the lateral side and decreasing the contact pressure on the affected medial cartilage, can provide an adequate mechanical environment for preventing further degeneration of the articular cartilage [28]. A considerable number of studies have demonstrated that HTO can provide pain relief and delay the need for TKA in patients with medial compartment OA of the knee [29]. The 10-year survival rate of patients undergoing HTO has been reported to be 74–89% [30]. During HTO, tourniquets are often used to reduce intraoperative bleeding, resulting in a clearer surgical field and reduced transfusion rates [3]. However, many studies, especially those related to TKA [31, 32], have questioned the safety and effectiveness of tourniquets. However, there are still no relevant studies reported in HTO. In addition, tranexamic acid has become the standard protocol for perioperative blood management in HTO and has been reported to significantly reduce intraoperative and postoperative blood loss without increasing the risk of complications. Therefore, whether the usage of tourniquets is necessary and increases the risk of complications under modern tranexamic acid regimens needs to be investigated.

The primary purpose of tourniquet use is to reduce intraoperative bleeding, thereby reducing transfusion rates and the risks associated with transfusion. We found that intraoperative blood loss in the non-tourniquet group was significantly higher than that in the tourniquet group, consistent with the literature [33, 34], although the difference was relatively small. However, the total blood loss did not differ significantly between these two groups, and none of the patients in either group required blood transfusion. Therefore, tourniquet use does not appear to be clinically significant in reducing perioperative blood loss and transfusion rates in HTO. This may be because, although a tourniquet can reduce intraoperative bleeding, release of the tourniquet can lead to activation of fibrinolysis, resulting in increased hidden blood loss [35]. In addition, the application of TXA itself can effectively reduce intraoperative and postoperative bleeding in HTO, and the effects of tourniquet inflation and deflation on the coagulation system may interfere with the haemostatic mechanism of TXA [36], resulting in the haemostatic effect of combined application of TXA and tourniquet not being superior to that of TXA alone. Similarly, Schnettler et al. found that total blood loss was highest when tourniquet alone was used during TKA, followed by TXA and tourniquet together, and lowest when TXA alone was used without tourniquet [13].

The benefits of using a tourniquet include improved surgical visibility and better visualization of the soft tissue and bone structure around the osteotomy site [37], which contributes to a more efficient and shorter procedure. Since surgical visibility is a subjective indicator that is difficult to accurately quantify, operation time and number of radiological exposures were selected as objective indicators to evaluate surgical visibility in our study. We found no significant difference in surgical duration or intraoperative fluoroscopy times between the tourniquet group and the non-tourniquet group. There was no significant difference in postoperative lower limb alignment between the two groups. In addition, the incidence of fractures of the lateral tibial cortex was similar in the two groups, with the tourniquet group being 18.75%, and the non-tourniquet group being 13.33%, which is consistent with the incidence (8.3–33.3%) reported in the literature [38, 39]. Therefore, the results of our study suggested that abandoning the routine use of tourniquets in open wedge HTO did not affect the surgical efficiency.

In fact, many studies have shown that tourniquet use is associated with higher pain scores, increased analgesic requirements and delayed recovery and rehabilitation in the early postoperative period [21, 40]. Notably, we found that the VAS pain scores in the non-tourniquet group were significantly lower than those in the tourniquet group at 1 and 2 days after surgery. Meanwhile, the early postoperative knee ROM of the former was significantly better than that of the latter. This suggests that routine use of tourniquets may be detrimental to early postoperative pain relief and functional recovery. Consistent with our study, a randomized controlled trial conducted by Huang et al. [41] found that abandoning routine use of tourniquets in TKA resulted in better functional recovery and lower postoperative pain. The possible reason for this phenomenon is that the ischaemia-reperfusion injury caused by tourniquet application leads to local oxidative stress and an inflammatory response [42], which aggravates the pain and swelling of the knee, and affects ROM and functional recovery in the early postoperative stage in HTO. However, in our study, the effect of tourniquet use on postoperative pain and functional recovery was limited to the early postoperative period, and this effect disappeared at the 3-month follow-up.

Moreover, several studies have demonstrated that tourniquet use during TKA may increase the risk of thromboembolism [43, 44]. A meta-analysis by Jiang et al. [33] showed that the incidence of DVT in patients using tourniquets was 25.3% after TKA, while that in patients without tourniquets was 17.7%. However, our results showed that no symptomatic DVT or PE occurred in either the tourniquet or non-tourniquet group in HTO with modern tranexamic acid protocols. The relatively short duration of the operation, the relative youth of the patients, and postoperative thrombotic prophylaxis, as well as early postoperative functional exercise and partial weight-bearing walking may contribute to reducing the incidence of thromboembolism. In addition, it has been reported that increased hidden blood loss caused by tourniquet application can lead to haematoma formation at the osteotomy site, resulting in incision complications [45]. However, in our study, only one patient in the tourniquet group had delayed wound healing, but the wound healed well at the 3-months follow-up. Of note, although our study did not find a difference in the incidence of overall complications at 3 months after surgery between the tourniquet and non-tourniquet groups, this may be related to the small sample size and short follow-up period.

The study has several limitations. First, this study was conducted at a single centre, and all surgeries were performed by the same surgeon, which minimizes confounding factors but does not facilitate the generalization of results to a larger population. Second, although we attempted to minimize selection bias, the retrospective nature of this study must be taken into account in drawing conclusions. Third, this study primarily focused on perioperative blood loss, early functional recovery and complications. However, extended follow-up is necessary to further assess the long-term effects of tourniquet use. Finally, our study was underpowered on rare endpoints, such as incision complications and symptomatic thromboembolic events. Future randomized controlled trials with larger sample sizes are needed to provide more robust conclusions regarding this topic.

## Conclusions

Forgoing tourniquet use does not increase perioperative total blood loss or the risk of complications in open-wedge high tibial osteotomy with modern tranexamic acid protocols, but benefits early recovery of knee function by reducing pain and increasing range of motion.

## Data Availability

The datasets used and/or analysed during the current study are available from the corresponding author on reasonable request.

## Abbreviations

HTO: High tibial osteotomy
OA: Osteoarthritis
TXA: Tranexamic acid
DVT: Deep venous thrombosis
PE: Pulmonary embolism
BMI: Body mass index
ASA: American Society of Anaesthesiologists
Hb: Haemoglobin
Hct: Haematocrit
ROM: Range of motion
VAS: Visual analogue scale
KSS: Knee Society Score
MPTA: Medial proximal tibia angle
HKA: Hip-knee-ankle angle
LOS: Length of stay
